# POWERS_*for*ID_: Personalized Online Weight and Exercise Response System for Individuals with Intellectual Disability: study protocol for a randomized controlled trial

**DOI:** 10.1186/s13063-017-2239-2

**Published:** 2017-10-23

**Authors:** William H. Neumeier, Nichole Guerra, Mohanraj Thirumalai, Betty Geer, David Ervin, James H. Rimmer

**Affiliations:** 1 University of Alabama at Birmingham/Lakeshore Foundation Research Collaborative, 331 School of Health Professions Bldg. 1705 University Blvd, Birmingham, AL 35294-1212 USA; 2 The Resource Exchange, 6385 Corporate Drive, Suite 301, Colorado Springs, CO 80919 USA

**Keywords:** Weight loss, Intellectual disability, Telehealth, Motivational interviewing

## Abstract

**Background:**

Intellectual disability (ID) is characterized by limitations in intellectual functioning and adaptive behavior. Adults with ID exhibit higher rates of obesity and poorer health status compared to the general population. Continuity of care and barriers to health-related activities may contribute to the poorer health status observed in this population. To address this problem, a tailored weight management online health information and communication technology platform, known as POWERS_f*orI*D_, was developed and is being tested to determine if this delivery mechanism can improve weight maintenance/weight loss in adults with ID.

**Methods:**

Obese adults with mild-to-moderate ID (n = 70) are randomized to the POWERS_*for*ID_ intervention or control group for a 24-week trial. Each group undergoes an assessment that includes body weight, waist circumference, and percent body fat at baseline and at weeks 6, 12, and 24. Physical activity barriers, healthy eating barriers, food frequency, and psychosocial wellbeing are measured at baseline and at weeks 12 and 24. Blood lipids are assessed at baseline and 24 weeks. Participants randomized to POWERS_*for*ID_ receive access to the POWERS_*for*ID_ website and calls from a health coach (weekly during weeks 1–12, biweekly during weeks 13–24). The health coach employs motivational interviewing techniques adapted for individuals with ID to promote behavior change. Participants randomized to the control group receive standard clinical weight-loss care. Differences in weight, waist circumference, blood lipids, percent body fat, and psychosocial self-report will be assessed. Barriers and facilitators of implementation as well as perception of study outcomes will be conducted via qualitative analysis.

**Discussion:**

POWERS_*for*ID_ is a novel information and communication technology platform designed to address health needs for adults with ID. This article describes the development and components of POWERS_*for*ID_. The overall aim is to assess usability and feasibility of POWERS_*for*ID_ for promoting weight loss for obese adults with ID over the course of a 24-week randomized control trial.

**Trial registration:**

Clinicaltrials.gov, NCT03139760. Registered on XXX

**Electronic supplementary material:**

The online version of this article (doi:10.1186/s13063-017-2239-2) contains supplementary material, which is available to authorized users.

## Background

Intellectual disability (ID) is characterized by significant limitations in intellectual functioning (IQ of 75 or less) and adaptive abilities, such as conceptual, social, and practical skills. An individual may be diagnosed with ID after assessment via standardized test batteries and structured interviews. Classifications of ID include mild (IQ 50–69), moderate (IQ 35–49), or profound [1]. Individuals with ID are more likely to be overweight or obese, have poorer health status, and limited access to health promotion programs compared to the general population [2–7]. The consequences of obesity predispose adults with ID to a greater risk of secondary health conditions that can impair their health status and quality of life [8]. Lack of healthy eating habits and regular physical activity may be associated with the higher rate of obesity seen in this population [9, 10]. However, current approaches to creating healthier lifestyles (i.e. improved nutrition and increased physical activity) for adults with ID can be extremely challenging because of many different barriers including financial constraints to join a fitness facility, transportation issues, limited choices, and the need for greater involvement and support from caregivers [11–15].

The current healthcare delivery system for adults with ID provides little guidance in health promotion to families or developmental disability service providers. As a result, many if not most individuals and care providers/families are unprepared to manage or improve weight management for people with ID [12]. General recommendations to get more exercise or eat better (e.g. restrict caloric intake, consume more fruits and vegetables, etc.) are often ineffective, particularly in populations where there are interactions between the environment, living arrangements, and supervised care. Compared to people without ID, people with ID experience much poorer continuity of care and health maintenance including receiving fewer routine and preventive health services such as blood pressure checks and cholesterol and cancer screenings [16]. Thus, few clinically successful weight-loss studies have been reported in the ID literature.

Individuals with ID and caregivers may be able to benefit from a tailored health information and communication technology (ICT) infrastructure that would allow greater continuity among staff and families in tracking and managing obesity in adults with ID. Online physical activity and nutrition guidance systems hold promise in assisting with recognition of what barriers must be overcome and strategies developed to reduce obesity rates in adults with ID [12]. ICT systems also allow for increased monitoring, one of the key elements of managing and reducing body weight [17, 18]. Likewise, these new technologies provide an opportunity to promote consistent healthcare delivery to individuals with ID, as they are using Internet and communication devices at rates similar to the general population [19].

To our knowledge, only one study incorporated the use of technology to assist with achieving weight loss in individuals with ID [20]. Researchers provided study participants with tablet computers for two months to assist with tracking physical activity, dietary intake, and video conference with a registered dietitian. On average, participants used the tablet for tracking dietary intake on 83% of days during the two-month period and lost 3–4% of body weight. The results of the study indicate the feasibility of individuals with ID to use ICT for health-related behaviors.

To date, there are no randomized controlled trials examining the effectiveness of a weight-loss program using ICT and motivational interviewing in obese adults with ID. Given the complex health needs and extreme fragmentation of healthcare delivery experienced by adults with ID, there is a strong and growing need to develop a scalable health IT system that can support the diverse needs of adults with ID. Therefore, the aim of this paper is to describe the design of a blended online health promotion intervention using an innovative e-Health platform that has been designed specifically for adults with ID to reduce modifiable clinical, anthropometric, and behavioral risk factors associated with obesity.

## Methods/Design

The intervention presented in this manuscript is based on two previous studies that reported successful weight loss using an online ICT weight management system with remote access to a health coach to reduce obesity in adults with physical disabilities and chronic health conditions [21, 22]. Results indicated statistically significant weight loss in both studies in the range of 3–7% [21, 22]. The system, referred to as POWERS (Personalized Online Weight and Exercise Response System), is a novel, multifocal-centered tailored intervention utilizing an innovative online tool designed to facilitate improvements in physical activity and nutritional behaviors.

The POWERS interface has been adapted to address the specific needs of adults with ID and their caregivers. In addition, health coaching with motivational interviewing (MI) techniques was adapted for this population [23]. The description of the study protocol follows the SPIRIT 2013 checklist, CONSORT statement for randomized trials, and TIDieR checklist for intervention description and replication [24–26].

### Study objectives

The overall study objective is to assess the usability, feasibility, and outcomes of a personalized online remote weight-management MI coaching system designed for use with adults with ID, which is referred to as POWERS_*for*ID_. The steps taken to achieve this overall objective include: (1) assessment of the acceptability and usability of the POWERS system for individuals with ID; (2) adaptation of the POWERS system based on qualitative feedback from participants, caregivers, and research staff; and (3) development of a randomized controlled trial (RCT) to examine the effect of POWERS_*for*ID_ on health status and body weight in obese adults with ID when compared to usual care.

### Setting and target population

Individuals with mild or moderate intellectual disability residing in and around Colorado Springs, CO, USA who receive healthcare services from a clinic specializing in developmental disabilities are eligible for the study. Only patients diagnosed with intellectual or developmental disability (IDD) are enrolled at the clinic. The clinic’s medical team determines patient eligibility to enroll at the clinic based on a diagnosis of an intellectual or developmental disability after reviewing available patient records (e.g. medical history, psychological evaluation, school records, developmental disability determination form, etc.). If patient records are limited or unavailable, then clinic staff providers perform medical evaluations to determine eligibility to enroll in the clinic. Eligibility to participate in the study is partially determined by the investigator’s observation of the individual’s functional skills (e.g. communication, ability to follow instructions with assistance, ability to use a computer with assistance, etc.). Ethical permission was obtained through the Institutional Review Board at a major university associated with the study design. All participants provide informed consent before participating. Eligible participants reside in either a group home, host home, family home, or independently. In compliance with ethical guidelines, participants without a legally authorized representative are also included. Individuals with ID who meet the following criteria are eligible for the study: (1) body mass index ≥ 30 kg/m^2^; (2) diagnosis of mild or moderate ID [1]; (3) age 25–50 years; (4) medical provider approval to participate in a weight-loss program; (5) has a regular caregiver willing to participate in a support role unless the participant serves as own legal guardian; and (6) has access to a computer with Internet throughout the week. Exclusion criteria include: (1) already participating in a weight-loss program; (2) a medical condition that prevents safe participation; and (3) insufficient capacity to consent.

### Intervention plan

**Fig. 1:**
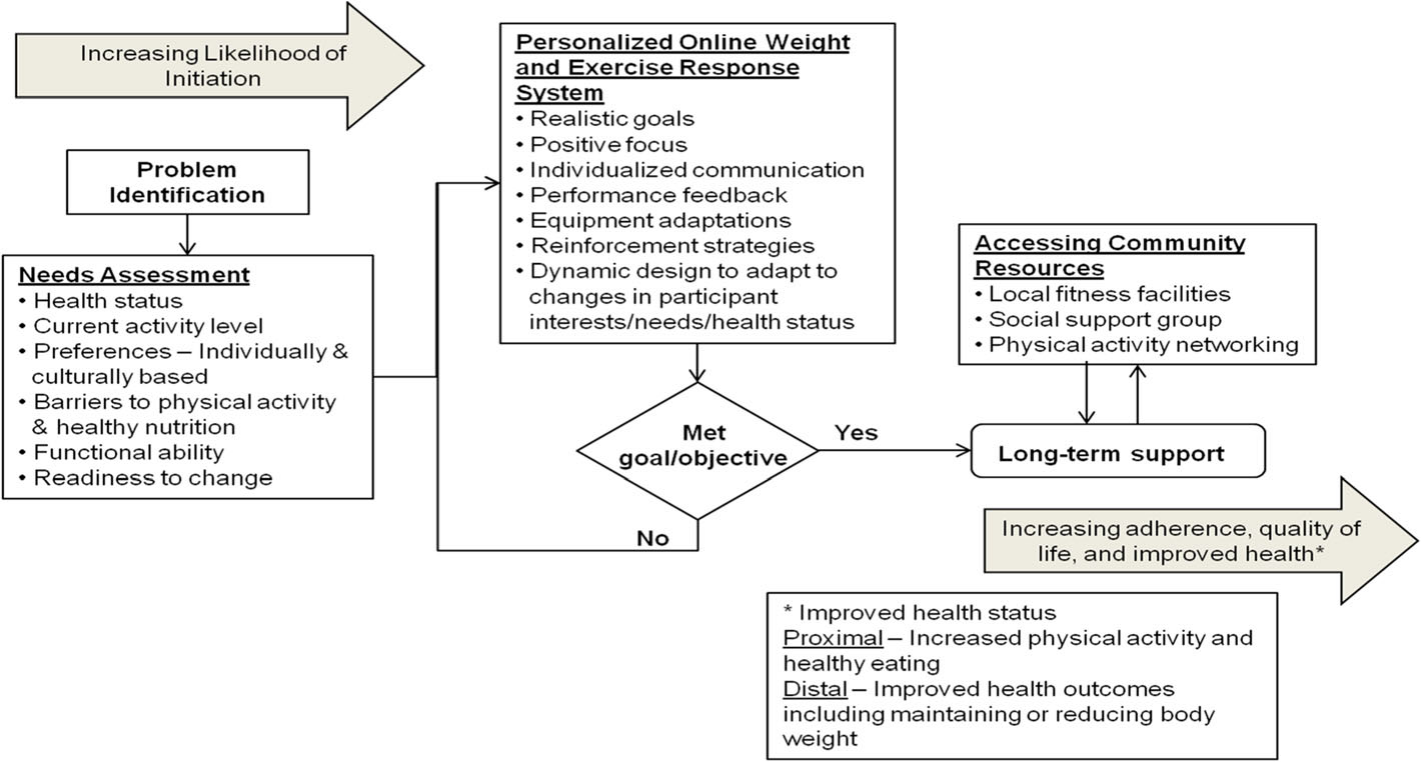
POWERS conceptual framework

The intervention plan (Fig. 1) was based on a conceptual model that has been previously validated [21]. The POWERS conceptual model combines approaches from other well-established models including the transtheoretical (stages of change model), person-centered theory, and socio-ecological model. The transtheoretical model is utilized at the Problem Identification stage to assess individual barriers, status, and preferences. The POWERS model also utilizes a person-centered approach to identify personal areas of change. The socio-ecological model is used for identifying community-based resources to assist with sustaining health-related behaviors. Starting from the left, the conceptual model begins with problem identification and needs assessment accomplished through a detailed evaluation of the individual’s health status, current activity level, needs and interests, eating behavior, personal and environmental barriers to physical activity, functional ability, and readiness to change. This detailed evaluation is referred to as the Health Appraisal Profile (HAP). The HAP is used to prepare a personalized exercise and nutrition program (middle column of Fig. 1), which includes goal-setting, positive focus, individualized communication and performance feedback, and adapting strategies to help the participant and/or caregiver overcome personal and environmental barriers. Attainment of goals can then be re-evaluated and adjustments made during coached portions of the intervention. The online platform also integrates local community resources, such as accessible exercise facilities and farmers’ markets. Community resources are populated by the POWERS system, the coach, or participant. These resources may assist the participant and their caregiver with accomplishing incremental personal objectives towards long-term maintenance of a healthy lifestyle. Overall, the model illustrates how individuals are guided through problem identification, behavior assessment associated with diet and exercise, and finally, long-term support to improve health and quality of life.

### POWERS_*for*ID_ platform and website

POWERS_*for*ID_ was customized specifically for adults with ID and is based on the original POWERS system. POWERS_*for*ID_ generates nutrition, exercise, health behavior, health education, and adapted disability-related content from a pre-populated database. Information generated from the participant’s HAP responses is customizable by the health coach. For example, a participant may report low intake of fruits and vegetables due to “dislike of diet and health food.” POWERS_*for*ID_ would then query the database and populate suggestions such as “add fruit to cereal, granola, or yogurt” or “make a fresh fruit smoothie.” The platform also identifies nearby stores where fruits may be purchased and links to a national website that has a smoothie recipe. The health coach also has the ability to freely customize and edit the recommendations (i.e. tips) based on new information and/or participant needs. If additional resources are needed, POWERS_*for*ID_ is able to incorporate local and national resources that may be utilized by the participant and coach. For resources outside POWERS_*for*ID_, links are provided to health resources hosted on the National Center on Health, Physical Activity and Disability’s (NCHPAD) website (www.nchpad.org). An eight-step process for identifying and targeting participant strategies is presented in Table 1.

**Table 1:** POWERS process for identifying and targeting participant health behavior strategies

Step	Description
1. Progress monitoring (by coach)	After the initial HAP evaluation, participants can access POWERS to log their progress in terms of weight, physical activity (type, duration, frequency), and diet (calorie intake, fat gram intake, and fruit/veg. servings) anywhere 24/7. During each coaching session, a health coach starts with the review of a participant’s progress since last session. The tracking data are displayed in interactive charts facilitating easy detection of progress or areas of concern. If the target BMI goal is reached, the coaching exercise is complete and the participant is referred to a community-based program; otherwise, the main POWERS steps below will be implemented. If the participant has significant health risk issues that are identified during monitoring, the participant will be advised to contact his/her physician.
2. HAP updates (by coach)	A health coach and a participant work together on the phone to update the HAP, a comprehensive assessment tool that includes a participant’s disability status (e.g. health conditions and functional ability), behaviors (e.g. physical activity, nutrition, screen time), and barriers to achieving diet and physical activity health promotion goals (e.g. separated into intrapersonal, interpersonal, community, and organizational barriers).
3. Objective selection (by POWERS)	A large set of “rules,” developed specialists in nutrition, exercise, health behavior, health education, and disability, are used to associate each wellness objective with specific disabilities, demographics, health issues, and a variety of conditions assessed in HAP. When one or more of these HAP conditions is met, the associated objectives are triggered and presented to the coach. For instance, if an adult participant has a BMI ≥ 30, the objectives of “lower calorie intake,” “increase physical activity,” etc. will be triggered because they are associated with the conditions “adult” and “BMI ≥ 30.”
4. Objective calibration (by coach)	The recommended wellness objectives from Step 3 are generic in nature. They need to be refined and calibrated to accommodate the special needs for the participant and be “measurable.” For instance, it is difficult to quantify the progress against a generic objective such as “lower calorie intake.” Instead, the coach and the participant should work together to set a more measurable objective such as “lower calorie intake to 2000 calories per day in 4 weeks.”
5. Strategy selection (by POWERS)	Similar to how wellness objectives are recommended from the POWERS knowledge base, strategies to achieve these objectives are also available in the POWERS knowledge base and triggered by matching HAP conditions. For instance, if “increase intake of fruits/vegetables” is selected as the wellness objective and the participant has indicated “dislikes diet or health food” as a nutrition barrier in HAP, strategy recommendation such as “add fruit to cereal, granola, or yogurt” and “make a fresh fruit smoothie” will be triggered.
6. Strategy enrichment (by POWERS)	POWERS further “enriches” the recommended strategies by providing a set of wellness education resources (both text materials and videos) to help implement the recommended strategies. These NCHPAD educational resources are associated with strategy recommendations in POWERS via keyword searches, instead of directly linked to the strategies, to provide the flexibility for NCHPAD staff to update these educational resources independently of the POWERS operations.
7. Strategy localization (by POWERS)	POWERS can also propose local community resources to help implement a specific wellness strategy. For instance, when proposing to play golf as a way to increase physical activities, POWERS can automatically search in its community resource database for golf courses that have accessible golf carts based on the locations (e.g. home, work, school, etc.) provided by the participant in HAP. POWERS can then display them on a Google Map using Google Map API. To allow POWERS coaches at local communities to maintain and share the information about these local resources themselves, POWERS allows coaches to submit information about these local resources and the system can automatically process them, including geocoding, to make them accessible to Google Map.
8. Strategy calibration (by coach)	Similar to how wellness objectives are calibrated for individual participants, strategies are refined and calibrated to be measurable and realistic. Instead of using the generic system-generated strategies such as “make a fresh fruit smoothie,” the telehealth coach and the participant could revise it to “make 2 fresh fruit smoothies every week” as the actual strategy. Also, note that Steps 6 and 7 above could provide additional support information to help implement the strategy, such as possible recipes from Step 6 and where to buy the ingredients from Step 7 on a map.

### POWERS_*for*ID_ website

POWERS_*for*ID_ includes a multifocal tailored website designed to facilitate improvements in physical activity and nutritional barriers. The website provides each participant with a personal space to monitor and track health related behaviors. The website also provides discussion boards to interact with a health coach and individualized tips for overcoming nutrition- and physical activity-related barriers. Each participant receives a unique username and login for the website. Once a participant is logged in, he/she sees an overview page that presents the latest message from the health coach as well as information related to weight-loss progress, physical activity goals, and nutrition goals. The participant also has access to areas focused on specific health behaviors. Specifically, there are separate sections in POWERS_*for*ID_ to track water consumption, food intake, and physical activity. Each of these pages reflects the participant’s overall progress towards a daily goal and displays weekly measures of progress. In addition, each page has specific tips for the participant and utilization of these tips as described in Table 1. The participant may also record daily behaviors in the “journaling” section. The health coach has the ability to view information logged by the participant. The coach may also make notes that are not viewable to the participant. These notes may serve as a reference for phone sessions between the participant and coach. A screenshot and description of the POWERS_*for*ID_ main page is presented in Additional file 1.

### Feedback on POWERS_*for*ID_

Qualitative feedback was obtained from five adults with ID and their caregivers over a six-week period. The original POWERS_*for*ID_ diet protocol emphasized a low carbohydrate diet, which has been demonstrated as effective for weight loss and loss of fat mass [27]. However, caregivers repeatedly emphasized difficulty with tracking carbohydrate intake for adults with ID. Caregivers also expressed concern that attempts to reduce carbohydrate intake intruded on the adult with ID’s autonomy. Based on this feedback, a targeted food system was piloted and implemented in place of carbohydrate tracking. The targeted food system utilizes information from a food frequency questionnaire and motivational interviewing techniques to allow the participant with ID to identify foods and dietary strategies he/she is willing to target and improve upon. The targeted food system also simplifies diet-related behaviors by shifting emphasis to only two to five targeted foods instead of relying on total carbohydrate intake. Other changes included simplification of the POWERS_*for*ID_ interface, specifically by eliminating information displayed in line graphs and instead utilizing a traffic light graphic to categorize foods. Infrastructure improvements were also implemented to increase usability for the health coach.

### Intervention

The study design is a 24-week RCT to assess the efficacy of POWERS_*for*ID_ on weight loss and overall health status (target sample: n = 70). The primary aim of this study is to test the feasibility and efficacy of the POWERS_*for*ID_ intervention in obese adults with ID. With a moderate effect size of 0.62 and a two-sided alpha of 0.05, a sample size of 35 participants per group will achieve a power of 0.80. After consent, all participants complete baseline measures and are then randomized to one of two conditions: (1) POWERS_*for*ID_ intervention; or (2) control. De-identified patient demographic data were used to conduct simulation checks to ensure balanced group allocation and to determine a proper randomization protocol. Simulation checks accounted for age, race, and ethnicity. After the simulation checks, a simple randomization sequence was developed. Each individual randomization assignment was then sealed in an envelope. Participants have equal probabilities of randomization to the intervention or control group. When it is time to randomize a participant to either arm, the health coach opens the next envelope in the sequence to reveal the group assignment. Participants are recruited, enrolled, and randomized by the health coach; all other research staff have been blinded to randomization. Participants randomized to the control group receive usual care from the clinic and related clinic staff. In addition, during the 24-week intervention, control group participants complete assessments at baseline, and at weeks 6, 12, and 24. Upon completion of the study period, control group participants are provided educational materials from an online information and resource center specializing in health and disability, as well as the option to contact a toll-free number and speak with an information specialist for further advice on physical activity and nutrition.

**Fig. 2:**
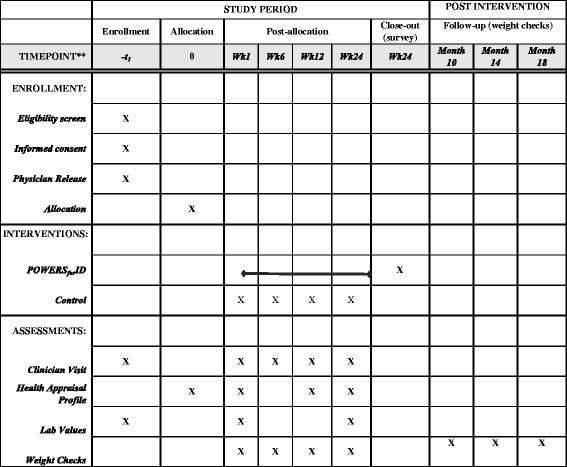
SPIRIT 2013 diagram for study protocol and outcomes

Participants randomized to the intervention group receive access to the POWERS_*for*ID_ website and review the interface with the health coach. The participant and coach schedule an initial phone call to review strategies to improve physical activity and diet. Subsequent phone calls occur weekly until the 12th week of the intervention and biweekly during weeks 13–24. All participants visit the clinic at baseline and at weeks 6, 12, and 24. The study protocol is summarized in Fig. 2 (based on SPIRIT 2013 guidelines).

### Intervention components

#### Health Appraisal Profile (HAP)

The HAP consists of demographics, reporting of primary and secondary disabilities, physical activity level, physical activity barriers, eating behaviors, food frequency, and psychosocial questionnaires [28, 29]. The HAP is administered to both the intervention and control groups at baseline then again at weeks 12 and 24. The HAP is completed by the participant with ID with assistance from the caregiver (if applicable) and health coach.

#### Goal setting

After the participant and caregiver (if applicable) complete the baseline HAP, the individualized (automated) goals and objectives are generated by the POWERS_*for*ID_ system and reviewed by the health coach before the first online meeting and phone call with the participant. After the health coach and the participant agree to the physical activity, nutrition and behavioral reinforcement plan, the project coordinator arranges the first coaching call. The initial call lasts approximately 30–60 min and is used to review the goals and implementation strategies selected by the POWERS_*for*ID_ system (established from the baseline HAP) and to set a schedule for the weekly (first 12 weeks) and biweekly (last 12 weeks) coaching calls, which last approximately 20–30 min. The goals and implementation strategies in physical activity and nutrition are finalized with the participant to ensure that they are consistent with the participant’s abilities, interests, and availability of community resources. Discussion points are noted by the health coach, permitting the research staff to review calls and monitor recommendations. During the calls, participants learn new strategies to improve their nutrition and physical activity behaviors (e.g. viewing a video of a certain activity, learning about healthy food choices) and are able to check the website with a password protected ID at any time for updates on their progress. Caregivers also have access to the system to view the progress of their care recipient.

#### Physical activity

Physical activity goals are set at a minimum of 30 min of aerobic exercise per day for the first four weeks of the intervention and gradually increased to 60 min per day (can be done in one or more sessions per day) during weeks 5–24, as reviewed and recommended by the person’s physician. The 60-min recommendation is based on the American College of Sports Medicine’s physical activity guidelines [30]. The participant also has the option to document strength and flexibility exercises. Participants receive physical activity options based on their interest level, what is available in their community, and affordability. The health coach may also provide recommendations and assist the participant with problem-solving perceived barriers to physical activity. Additional resources and recommendations are auto-populated by POWERS_*for*ID_. Each participant is given a pedometer to cross-validate their self-reported physical activity level.

#### Nutrition

The participant and coach select the nutrition goals. During the initial phone call, the participant and coach review food suggestions populated to POWERS_*for*ID_ from the HAP food frequency questionnaire. The participant and coach collectively identify and discuss “problem” foods (i.e. foods high in fat, carbohydrate, and caloric value) and agree on strategies to reduce consumption of one or more of these foods. The participant is also given instructions on what constitutes a serving size for each food group, as well as proper methods for measuring foods. After agreeing on targeted foods, the health coach populates the food item(s) into POWERS_*for*ID_ along with their corresponding nutritional values. The participants then log their daily consumption of these target foods with assistance from their health coach.

#### Reinforcement system

The reinforcement elements located on the POWERS_*for*ID_ profile page include a set of symbols associated with completion and logging of a day’s physical activity and nutrition goals. Small incentives are mailed or given to participants when they meet their target “healthy” points for the week ($2 for attaining 100% of weekly goals and prorated accordingly for achieving a lower percentage or $1 if 50% of goals are achieved). Participants who are adherent to the protocol (based on their physical activity and nutrition scores) receive a phone call from the health coach to compliment them on having a successful week.

#### Coaching calls and motivational interviewing

Coaching calls are used to support participants and last approximately 20–30 min. The calls provide an opportunity to review participant progress, discuss barriers or setbacks, and identify future objectives. The calls occur weekly during the first 12 weeks of the intervention and biweekly in weeks 13–24. Each phone call is scheduled with the coach, participant and caregiver (if applicable) in advance. Prior to a phone call, the coach reviews any notes or discussion board posts as well as notes from previous phone calls and information logged by the participant on the POWERS_*for*ID_ website.

During the phone call, coaches utilize MI techniques adapted for individuals with ID [23]. MI has been demonstrated as efficacious for changing health behaviors [31]. MI typically involves abstract reasoning, but adaptations respectful of an individual’s cognitive level may still render motivational interviewing effective [23]. In addition, MI allows for person-centered interactions. The health coach provides guidance and suggestions but does not make decisions; it is up to the participant to decide on his/her best course of action. The coach’s use of MI techniques and participant response are analyzed via qualitative methods described below.

#### Additional materials

In addition to having access to the POWERS_*for*ID_ website and coaching calls, participants in the intervention group also receive additional materials to aid in study progress and behavior monitoring. These materials include a pedometer to monitor step count, flyers with information on logging into the website, a bathroom scale (only if the participant does not own an appropriate scale) with a guide to weighing themselves frequently, and nutrition handouts. Scales are to be returned once the participant has completed the entire study. These additional materials support the participant between coaching sessions as well as promote objective reporting.

### Outcomes

Outcome measures and timing of administration are listed in Table 2. Clinic visits include routine care, the HAP, and measurement of body weight and body composition (via waist circumference and bioelectrical impedance analysis). Blood samples for measurement of lipids and blood sugar are collected at baseline and week 24. Participants will be required to visit their medical provider at baseline and weeks 6, 12, and 24. During the visits, participants will receive medical supervision that mimics the routine care provided by the clinic. Routine care consists of measuring a patient’s blood pressure, 0_2_ saturation, urinalysis, height, weight, and waist circumference. Medical records will be accessed to collect the participant’s blood sample results. For the POWERS_*for*ID_ intervention clinicians will also measure body composition via bioelectrical impedance analysis and waist circumference. Standard procedures for assessing anthropometrics, such as removal of additional clothing items, measuring from between the lowest rib and iliac crest for waist circumference, will be utilized in line with standardized clinical procedures. The HAP is completed following completion of clinical measures and may be completed with assistance from a caregiver (if applicable) and/or the health coach.

**Table 2:** Outcome measures, instruments, and time points

Measures	Instruments	Time point(s)
Adherence	Logs, enrollment metrics, tracking data	Daily
Demographics	Demographics	Baseline
Functional abilities	POWERS_*for*ID_ disability, health and function	Baseline
Body weight	Scale and bioelectrical impedance analysis of body composition	Clinically measured at baseline and weeks 6, 12, and 24
Physical activity behaviors	Physical Activity and Disability Scale; Barriers to Physical Activity and Disability Scale; Exercise Perception Scale; Self-Efficacy Related to Exercise for People with DD Scale	Baseline and weeks 6, 12, and 24
Physical activity	Physical activity points and steps (pedometer)	Daily
Barriers to PA	Barriers to Physical Activity and Disability Scale	Baseline, weekly, 24 weeks
Nutritional behaviors	Block Food Frequency Questionnaire; Fruit and Vegetable Self-Efficacy Scale; Fruits and Vegetables Outcomes Expectancy Scale; Barriers to Fruits and Vegetables Scale	Baseline and weeks 6, 12, and 24
Nutrition	Healthy eating points	Daily
Dietary barriers	POWERS_*for*ID_ dietary barriers	Baseline and weeks 6, 12, and 24
Psychosocial	*HealthMatters* psychosocial questionnaire; Glasgow Depression Scale for people with a Learning Disability	Baseline and weeks 6, 12, and 24
Evaluation of POWERS_*for*ID_	Semi-structured interviews (qualitative analysis)	Post-intervention
Lipids	Blood test	Baseline and 24 weeks

#### HAP questionnaires

Questionnaires used in the HAP have been specifically designed for and previously utilized in populations with intellectual and developmental disabilities except for the Block Food Frequency Questionnaire. Questionnaires include: Physical Activity and Disability Scale [32], Barriers to Physical Activity and Disability Scale [33], Exercise Perception Scale [34], Self-Efficacy Related to Exercise for People with DD Scale [35], Fruit and Vegetables Outcome Expectations Scale [36], Fruit and Vegetable Self-Efficacy Scale [37], Barriers to Eating Fruits and Vegetables Scale [38], Glasgow Depression Scale for people with a Learning Disability [39], psychosocial questionnaires reproduced from the *Health Matters* curriculum – a health behavior curriculum for individuals with intellectual and developmental disability [28], and the Block Food Frequency questionnaire.

Semi-structured interviews are also obtained post intervention to gather qualitative data from intervention participants and caregivers. Three questions are used: (1) What barriers and facilitators were associated with implementing the recommendations? (2) What strategies were and were not effective in changing behavior? and (3) What additions would you recommend for the POWERS_*for*ID_ intervention?

### Fidelity

Participant fidelity is assessed based on participation in-clinic visits, phone calls, and website use. Fidelity for the research team includes scripts and objective cut-off measures for interacting with participants. There are set cut points for determining when a scheduled participant interaction is counted as missed. For example, if a participant and coach have a phone call scheduled but the participant has not answered by the third call, the coach discontinues calling. If the participant does not respond to the coach’s voicemail within two days then that week’s phone call is counted as missed. Also, the coach has a checklist to follow for each phone call. The project coordinator reviews call logs and clinic visit logs once a month to ensure adherence is correctly documented by the health coach. The project coordinator also randomly selects and reviews 20% of the call checklists. These fidelity protocols increase the project’s overall internal validity and replicability.

### Data analysis

Data analysis is performed by a project coordinator and statistician who are blinded to the intervention group assignment. Participant attrition, session attendance, and instrument completion are recorded and analyzed using descriptive statistics. We hypothesize that by the end of the study, 70% of the participants will be retained, 70% will participate in all coaching sessions, and 100% will have completed at least 80% of the survey instruments at each data collection point. Data will be analyzed with mixed effects models to assess the effect of POWERS_*for*ID_ on participant outcomes. A mixed model is a generalization of the standard linear model that permits repeated measures data to exhibit correlation and non-constant variability over time. We will use each outcome as a time-varying dependent variable and group (POWERS_*for*ID_ vs. control) as main fixed effects. Baseline demographics will be entered as time-invariant covariates if baseline group differences are observed. Intercept will be modeled as random effects. We hypothesize that the trajectory slope of participant outcomes will be more favorable in the POWERS_*for*ID_ group compared to the control group, which will result in a significant GroupXTime interaction effect. We will analyze our data using both on-treatment and intent-to-treat approaches. Participants lost through attrition will be compared on demographic and prognostic variables to those who remain in the study to examine the sample representativeness and potential treatment bias.

Qualitative data analysis from the POWERS_*for*ID_ study results will address motivational interviewing applications, barriers, and successes of the intervention. We will perform thematic analysis using ATLAS.ti software. Thematic analysis is an iterative process that involves the researcher as the instrument for: (1) coding qualitative data; (2) cross-comparing codes for pattern identification; (3) grouping codes into broader themes; and (4) interpreting thematic relationships [31, 40]. We will also report on the level of data saturation achieved.

## Discussion

The POWERS_*for*ID_ intervention aims to adapt and assess a novel telehealth interface that has been proven effective for other populations with disabilities. Overall, there are few publications demonstrating weight loss in adults with ID [41]. Recently, Ptomey et al. [42] reported clinically significant weight loss for adults with intellectual disability utilizing conventional diet recommendations (500–700 kcal/day restriction) in conjunction with monthly counseling sessions. Greater weight loss was observed in participants recommended a stoplight-based diet plus counseling. These findings support the effectiveness of stoplight-based information delivery, where “red” signifies foods and actions to avoid and “green” signifies foods and actions to be pursued.

After pilot assessment, the POWERS_*for*ID_ website and nutrition material were redesigned utilizing the stoplight theme. Ptomey et al. [20] also previously reported successful food logging and video chat sessions for adolescents with ID through use of a tablet computer, indicating technology may be effective with individuals with ID. To our knowledge, no interventions targeting weight management for individuals with ID utilizing an online platform and motivational interviewing techniques have been tested with this population. POWERS_*for*ID_ is the first intervention addressing diet and physical activity habits in adults with ID through an online interface and coaching calls. If the POWERS_*for*ID_ system is demonstrated to be efficacious, it could be incorporated into clinical health settings that have a similar specialty in providing healthcare services to adults with ID as the clinic involved in this study. A home-based telehealth approach would be helpful in assisting a population that is at greater risk of obesity and other chronic health conditions.

Preliminary findings from this study have identified several challenges. First, adults with ID may be unwilling to alter familiar routines or make changes to diet and physical activity habits. Food is often used as a reward in the ID population, and it is a challenge replacing unhealthy food choices (e.g. candy, cookies, chips, etc.) with healthier alternatives. Second, intervention materials have been adapted to the cognitive abilities of the target population, but concepts such as nutrition labels and serving size are difficult for some individuals to comprehend. Phone sessions and clinic visits will provide an opportunity for the coach to identify the individual’s abilities. With the assistance of the research team, the coach and participant can identify health-related behaviors that are effective for the individual and new solutions can be archived for future use. Third, individuals with ID and/or their caregivers may have other medical issues or stressors that increase difficulty of sustained weight loss. Coaches must be respectful of extraneous circumstances and develop solutions that avoid overburdening family members. For these reasons, the POWERS_*for*ID_ interface and the protocol’s time commitment has been simplified as much as possible. Preliminary feedback indicated the POWERS_*for*ID_ carbohydrate food log plus system interface were overly complex. Any health tracking requires additional effort on the part of the participant, although efforts have been made to reduce overall workload for the participant. Individuals enrolling in the POWERS_*for*ID_ study indicate a desire to be “healthier” and the goal of the system is to encourage health-related behaviors.

Notwithstanding the potential findings from this study, POWERS_*for*ID_ does provide a structure for carefully monitoring and managing key health behaviors in adults with ID. The system has the potential to raise their level of awareness about their own health behaviors as well as awareness of their caregiver and/or direct support staff when appropriate. Individuals with ID are in need of additional accessible health promotion, and the POWERS_*for*ID_ project aims to address this need. Final results from the intervention study are expected in 2018.

### Trial status

This trial is currently ongoing and open to recruitment. Recruitment began 20 September 2016.

## Additional file

Additional file 1: Screenshot and description of the POWERS_*for*ID_ homepage. (DOCX 98 kb)
