# Supplementary material for: POWERSforID: Personalized Online Weight and Exercise Response System for Individuals with Intellectual Disability: study protocol for a randomized controlled trial
Source: Trials. 2017 Oct 23;18:487. doi: 10.1186/s13063-017-2239-2 (PMC5653469; doi:10.1186/s13063-017-2239-2)

Additional File 1. Screenshot and description of the POWERS*_for_*_ID_ homepage.

The POWERS*_for_*_ID_ homepage loads immediately after a participant has entered his/her username and password. The homepage includes multiple points the participant can interact with, including a message from the health coach, a view of progress towards study incentives, tracking for weight, physical activity, and nutrition, and an area to exchange messages with the health coach. Across the top there are multiple tabs that the participant may click for more detailed information and tracking. The “Getting Started” tab leads to information on how to use the website and quick resources for nutrition and physical activity. The “Water,” “Food and Drink,” and “Exercise” tabs provides description of the participant’s daily and weekly goals in the related topic. Tips to assist the participant with achieving individualized goals are also presented on each of these pages. The “Journal” tab is where the participant logs his/her physical activity and selected food intake. The “Profile” includes the Health Appraisal Profile. Avatars and other information can be customized to the participant’s liking.


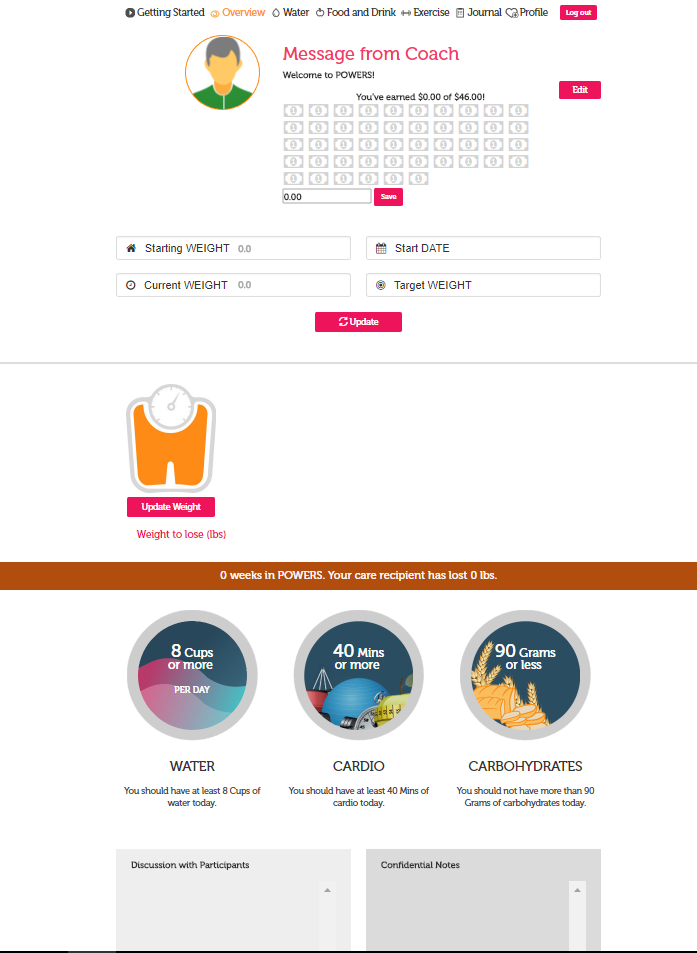

Supplement: Additional file 1: — Screenshot and description of the POWERSforID homepage. (DOCX 98 kb) [file 13063_2017_2239_MOESM1_ESM.docx]
